# The geography of COVID-19 vaccine completion by age in North Carolina, U.S.

**DOI:** 10.1371/journal.pone.0304812

**Published:** 2024-08-09

**Authors:** Hilary Sandborn, Paul Delamater, Noel T. Brewer, Melissa B. Gilkey, Michael Emch

**Affiliations:** 1 Department of Geography and Environment, University of North Carolina at Chapel Hill, Chapel Hill, North Carolina, United States of America; 2 Carolina Population Center, University of North Carolina at Chapel Hill, Chapel Hill, North Carolina, United States of America; 3 Department of Health Behavior, Gillings School of Global Public Health, University of North Carolina at Chapel Hill, Chapel Hill, North Carolina, United States of America; 4 Department of Epidemiology, Gillings School of Global Public Health, University of North Carolina at Chapel Hill, Chapel Hill, North Carolina, United States of America; Cleveland Clinic Lerner Research Institute, UNITED STATES

## Abstract

**Background:**

Geographic variation in COVID-19 vaccination can create areas at higher risk of infection, complications, and death, exacerbating health inequalities. This ecological study examined geographic patterns of COVID-19 vaccine completion, using age and sociodemographic characteristics as possible explanatory mechanisms.

**Methods and findings:**

Using 2020–2022 data from the North Carolina COVID-19 Vaccination Management System and U.S. Census Bureau American Community Survey, at the Zip code-level, we evaluated completion of the primary COVID-19 vaccine series across age groups. We examined geographic clustering of age-specific completion by Zip code and evaluated similarity of the age-specific geographic patterns. Using unadjusted and adjusted spatial autoregressive models, we examined associations between sociodemographic characteristics and age-specific vaccine completion. COVID-19 vaccine completion was moderately geographically clustered in younger groups, with lower clustering in older groups. Urban areas had clusters of higher vaccine completion. Younger and middle-aged groups were the most similar in completion geographically, while the oldest group was most dissimilar to other age groups. Higher income was associated with higher completion in adjusted models across all age groups, while a higher percent of Black residents was associated with higher completion for some groups.

**Conclusions:**

COVID-19 vaccination completion is more variable among younger age groups in North Carolina, and it is higher in urban areas with higher income. Higher completion in areas with more Black residents may reflect the success of racial equity efforts in the state. The findings show a need to reach younger populations and lower income areas that were not prioritized during early vaccination distribution.

## 1. Introduction

Vaccination is a safe and effective intervention for reducing SARS-CoV-2 infections and risk of complications and death from COVID-19. In the U.S., the first COVID-19 vaccine was approved under an emergency use authorization in early December 2020 and received approval for use by the Centers for Disease Control and Prevention (CDC) [1]. States managed their own COVID-19 vaccination rollout and eligibility programs. Early eligibility began with older populations, and distribution was primarily through mass vaccination facilities, which were more often located in wealthier, whiter, and more urban areas [2–4]. Later, vaccination was mandated or strongly suggested by various non-governmental entities, including health care facilities and universities [5]. The emergency-use approval, rollout and eligibility guidelines, and mandates for the COVID-19 vaccine produced a novel experience for U.S. residents and a vaccination landscape with marked variation in vaccine completion based on age, race and ethnicity, socioeconomic status, and geography [6–8]. Geographic variation in COVID-19 vaccination can create areas at higher risk of poor outcomes, exacerbating inequalities.

Few studies have examined geographic variation in COVID-19 vaccine uptake [6, 8, 9], and assessment of geographic patterns of uptake by age has been limited. Using a geographic perspective to study age-specific patterns could inform both geography-based and age-based vaccine interventions. Our study evaluated to what extent age-specific COVID-19 vaccine completion is geographically clustered, the similarity of age-specific geographic patterns of vaccine completion, and associations between sociodemographic characteristics and age-specific vaccine completion.

## 2. Methods

### 2.1 Unit of analysis and age strata

Our study focused on North Carolina, U.S, using Zip code as the unit of analysis. The age groups were 5–11, 12–15, 16–24, 25–49, 50–64, and 65+ years, mirroring those used for data reporting by the North Carolina Department of Health and Human Services (NCDHHS) and CDC [10]; they also reflect North Carolina’s eligibility criteria for older individuals (65+ years) early in the vaccination campaign [11] and age categories (5–11 and 12–15 years) mirroring later vaccine authorization [12, 13]. Children younger than 5 years were ineligible for vaccination during the study period [14].

### 2.2 Outcome data

The outcome was COVID-19 vaccine completion, operationalized as having received two doses of the Pfizer-BioNTech or Moderna vaccines, or a single dose of the Johnson & Johnson/Janssen (J&J) vaccine, further operationalized as the percent of Zip code residents by age group who had completed COVID-19 vaccination by May 31, 2022.

Vaccination data came from the COVID-19 Vaccine Management System (CVMS) maintained by NCDHHS. The CVMS is a dose-level database that includes a unique person identifier, as well as the recipient’s age, Zip code of residence, vaccine received, and dose number. Data cleaning included removing records without a valid North Carolina Zip code or identifier value and removing duplicates (S1 Appendix).

We identified individuals who had completed vaccination by extracting records from the CVMS labeled as a second dose of a Pfizer-BioNTech or Moderna vaccine or a first dose of the J&J vaccine. We used the unique person identifier to ensure that individuals were not double counted. For each Zip code, we summed the number of people who had completed vaccination for each age group (and for all residents regardless of age). To calculate the percent completely vaccinated for each Zip code, we divided the number of people completely vaccinated by the corresponding population derived from U.S. Census data.

### 2.3 Covariates

Covariates were variables associated with COVID-19 vaccine uptake in previous individual-level studies. They included Zip code-level median household income [15–21], race (% Black population) [15–19, 21, 22], and rurality [15, 16, 21] as variables of interest and gender (% female population) [15–17, 21, 22] and occupation (% healthcare workers) [23, 24] as control variables. Healthcare workers included all health diagnosing and treating practitioners, technologists and technicians, and care support occupations.

Census block group-level population counts by age category and socioeconomic characteristics (income, race, gender, and occupation) were collected from the U.S. Census Bureau’s American Community Survey (ACS) 5-year estimate for 2016–2020 [25]. Census block-level data with 2020 population counts, core based statistical area [26], county population-weighted centroids [27], and urban area [28] data used for calculating rurality were collected from the U.S. Census Bureau for the most recent year available. Zip code area boundaries and point locations were acquired from Esri [29, 30].

Although Zip codes can change over time and do not nest within U.S. Census geographies [31], they are commonly used in health-related analyses [32] and are the only geographic information that was made available in the CVMS. As such, additional data processing was required. First, we created a crosswalk to assign point-based Zip codes (e.g., those used for some Post Office [PO] Boxes or governmental entities) to the areal Zip code they are located within using a spatial join. In the outcome data, we assigned any resident of a point Zip code to their corresponding areal Zip code. Second, we used the areal interpolation with control zones approach [33, 34] to estimate Zip Code-level income, race, gender, and occupation data based on the block group-level data from the ACS. The areal interpolation method first required spatially joining 2020 census block population points the areal Zip codes; the block population data were then aggregated (summed) by block group and Zip code pairs (the population of each block group located in each Zip code). The population count for each block group and Zip code pair was divided by the total population of the corresponding block group to calculate the proportion of each block group’s population residing in each Zip code. The ACS block group-level attribute count data for income, race, gender, and occupation were then multiplied by the proportion for each corresponding block group and Zip code pair, essentially allocating the block group counts to the Zip code units. A final step included aggregating the allocated values by Zip code and converting the counts to proportions based on total population (occupation proportions were based on the population aged 16+ years, per the original data).

Zip code rurality was measured by calculating the index of relative rurality (IRR), a continuous measure that varies from 0 (most urban) to 1 (most rural) based on four dimensions: population size, population density, urbanization, and distance to the closest metropolitan area. We defined urbanization as the percentage of each Zip code region falling within a U.S. Census Bureau urban area and calculated distance to the closest metropolitan area as the Euclidean distance from the Zip code population-weighted centroid to the nearest central county population-weighted centroid within a metropolitan area. To calculate the IRR value, the rurality dimensions were rescaled and averaged per the original methodology [35].

### 2.4 Data analysis

Eight Zip codes were removed due to missing population or demographic information resulting in 756 of the 764 original Zip codes in the final analytic dataset.

We used Moran’s *I* to evaluate whether the age-specific (and overall) percentage of the population completely vaccinated was geographically clustered at the Zip code-level [36]. The metric is scaled from -1 to 1, with -1, 0, and 1 indicating perfect geographic dispersion, randomness, or clustering, respectively. The local indicator of spatial autocorrelation (LISA) was used to identify geographic clusters of high or low vaccination percent. We defined a high-high cluster as a Zip code with a high percent of the population completely vaccinated located adjacent to other Zip codes having high vaccination percentages and vice versa for low-low clusters [37]. LISA analysis also identified high or low outliers (e.g., a high-low outlier is defined as a Zip code with a high percent of the population completely vaccinated located adjacent to Zip codes with low vaccination percentages, and vice versa for low-high outliers). Because Zip codes are irregularly shaped geographic units, we defined spatial neighbors using Queen’s case connectivity with row standardization [38, 39]. Pearson’s correlation was used to evaluate the association of Zip code-level completion percentages among the age groups.

We used both unadjusted (bivariate) and adjusted (multiple) linear regression to analyze associations between sociodemographic characteristics and age-specific COVID-19 vaccine completion percentages (and overall completion percentage) at the Zip code-level. We initially used ordinary least squares (OLS) regression but found spatial autocorrelation in the regression residuals (all groups except 65+ years), necessitating spatial autoregressive models. We selected a spatial lag model over a spatial error model with a data-driven approach [40], despite the spatial error model being a closer theoretical fit for our process [41, 42]. Using a spatial autoregressive model extends the utility of a linear regression model by accounting for the spatial structure of the data [42]. Regression coefficients were standardized, allowing for comparison of the coefficients within and across models. As a sensitivity analysis, we used the spatial error model. Education, political affiliation, and ethnicity were initially included as predictor variables; however, due to multicollinearity, they were excluded (S2 Appendix). Analyses used two-tailed tests, with a critical alpha of 0.05. Analyses were conducted using ArcGIS Pro version 2.7.0 [43] and R version 4.2.0 [44].

### 2.5 Ethics statement

The University of North Carolina at Chapel Hill Institutional Review Board reviewed this study and determined it to be exempt (#21–1180, May 5, 2021).

## 3. Results

As of May 31, 2022, North Carolinians had received 16,265,469 doses of the COVID-19 vaccine with 5,978,837 people age 5+ years having completed vaccination (58% of the state population aged 5+). Summary statistics of Zip code-level vaccination completion percent and population characteristics are presented in Table 1.

**Table 1:** Descriptive statistics of Zip code-level vaccination completion and population characteristics (n = 756 Zip codes).

	Mean	Lower Quartile (Q1)	Median (Q2)	Upper Quartile (Q3)
**COVID-19 Vaccine Completion (%)**				
Overall (5+ years)	58.1	48.0	55.7	65.8
5–11 years	20.1	9.4	14.1	24.1
12–15 years	37.8	23.7	33.0	47.6
16–24 years	48.2	33.7	43.7	58.2
25–49 years	52.6	40.6	49.2	62.1
50–64 years	66.6	56.4	65.6	77.1
65+ years	84.0	75.8	86.3	100.0
**Covariates**				
Black population (%)	18.7	4.1	13.1	29.1
Female population (%)	50.8	49.3	51.2	52.8
Healthcare worker population (%)	5.3	3.3	5.1	6.9
Median household income ($)	56,984	44,897	52,528	63,612
Index of relative rurality (0 = most urban; 1 = most rural)	0.46	0.35	0.50	0.58

### 3.1 Geographic distribution and clustering

**Fig 1:**
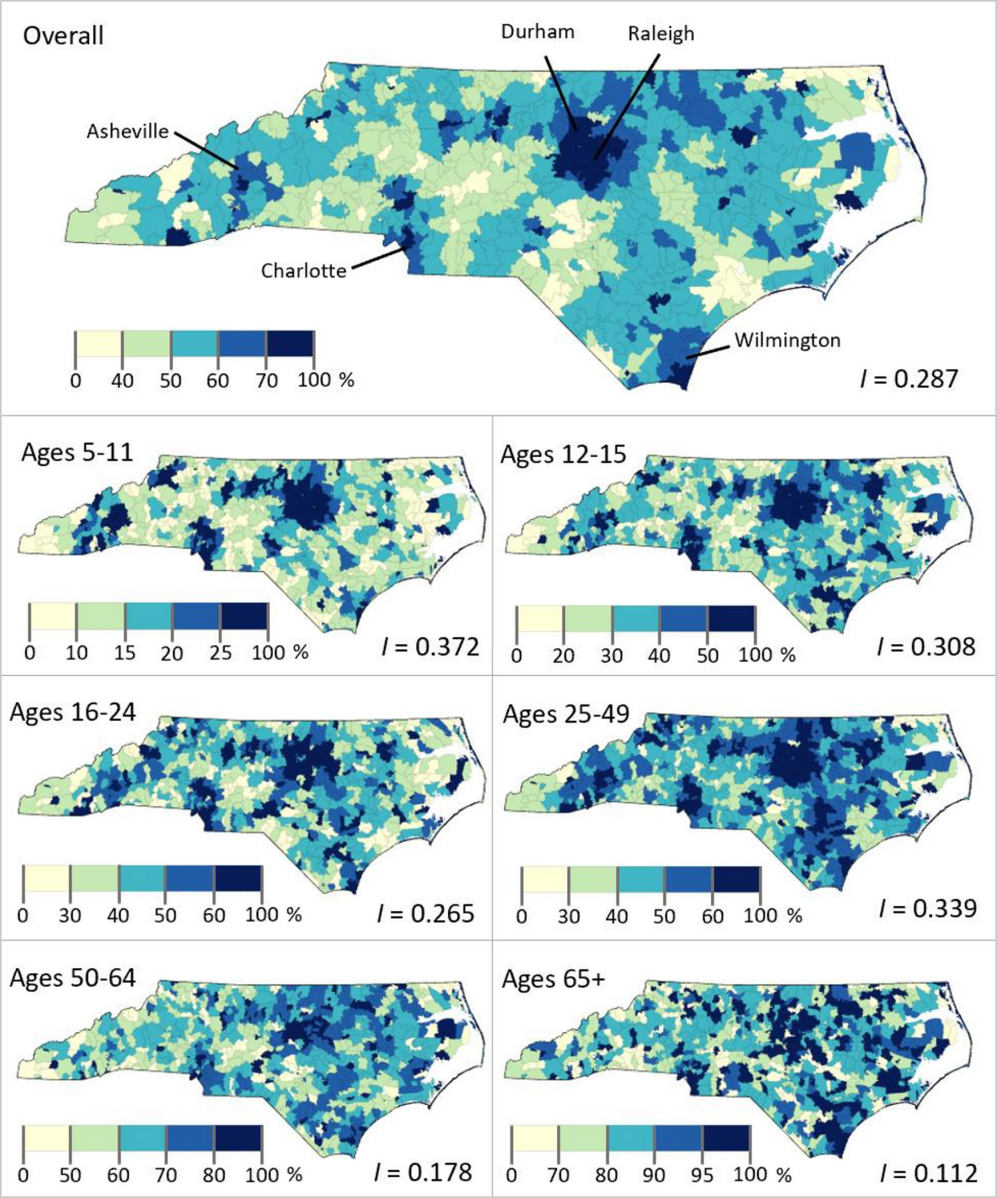
COVID-19 vaccine completion in North Carolina, U.S by Zip code. Shown as the percentage of the age group in each zip code with a complete vaccination status as May 31, 2022. The Moran’s I is for the vaccine completion percentage of each age group.

North Carolina’s urban areas, including Raleigh, Durham, Charlotte, Asheville, and Wilmington, had the highest percentage completely vaccinated for COVID-19 (Fig 1). The more rural regions of the state had relatively low completion percentage values. Regional patterns were more apparent for younger age groups.

Moran’s *I* values for vaccine completion percentage were positive across all age groups (all *p* ≤ 0.001), indicating geographic clustering (Fig 1). The magnitude of clustering was greater in younger age groups, ranging from moderate clustering in the age 5–11 group (*I* = 0.372) to slight clustering in the age 65+ group (*I* = 0.112).

**Fig 2:**
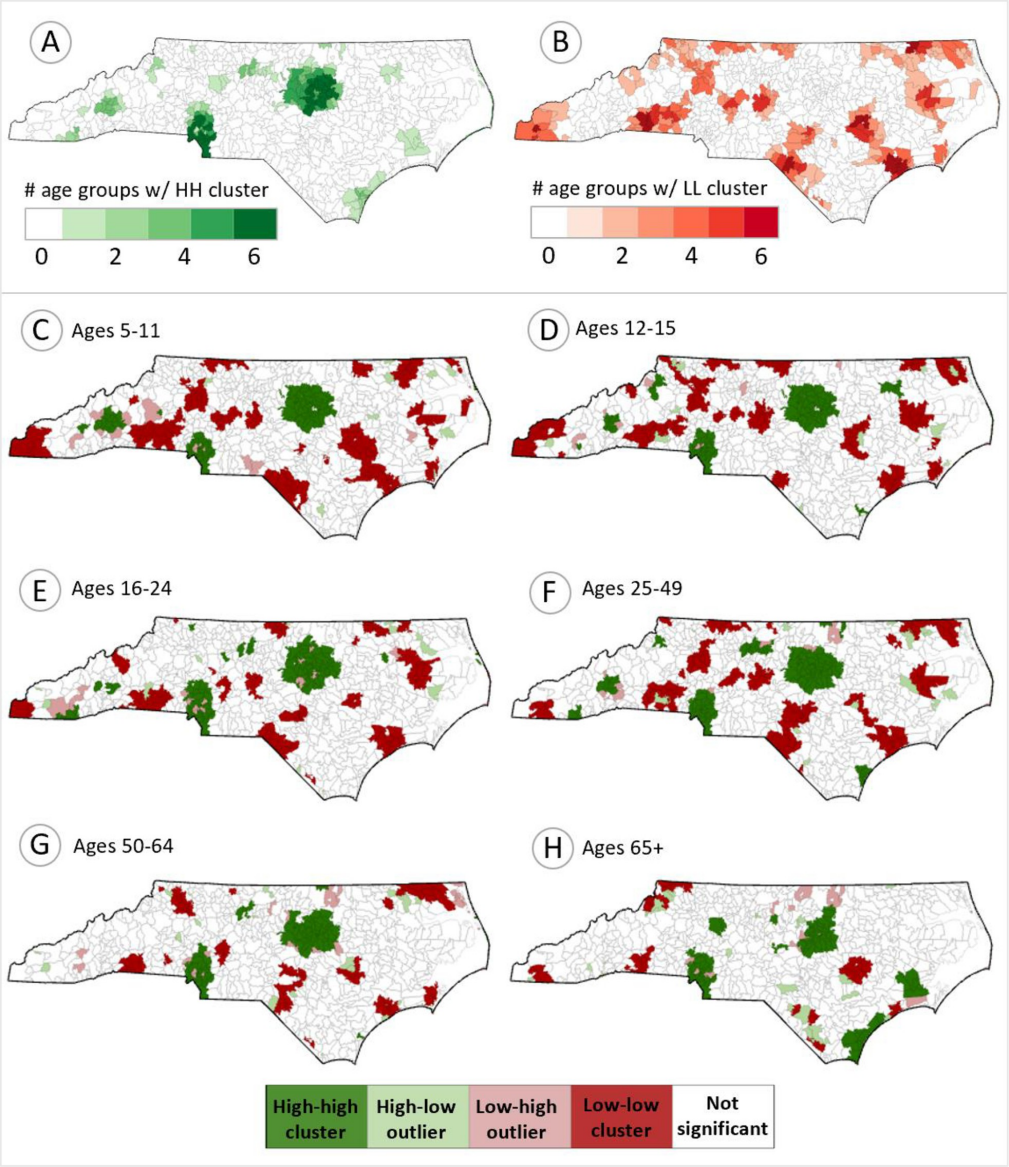
COVID-19 vaccine completion percentage clusters and outliers by Zip code (LISA results). Panels A and B show the number of age groups (out of 6) in which a Zip code was in a high-high (HH) cluster and low-low (LL) cluster of vaccine completion, respectively. Panels C-H present the age-specific LISA results for vaccine completion, showing HH and LL clusters, and high-low (HL) and low-high (LH) outliers for each group.

In the LISA analysis, the age 25–49 group had the largest number of Zip codes in high-high (HH) clusters (131), while the age 50–64 group had the fewest (96). For low-low (LL) clusters, the age 5–11 group had the most Zip codes (114), and the age 65+ group had the fewest (35), reflecting the groups’ overall completion percent. Regionally, HH clusters were consistently located in urban areas for most age groups (Fig 2). The region near Asheville had some HH clusters only for the four youngest age groups, while the Wilmington region had prominent HH clusters only for the age 25–49, and 65+ groups. LL clusters were identified in the more rural regions of North Carolina.

### 3.2 Similarity among geographic patterns of completion

**Fig 3:**
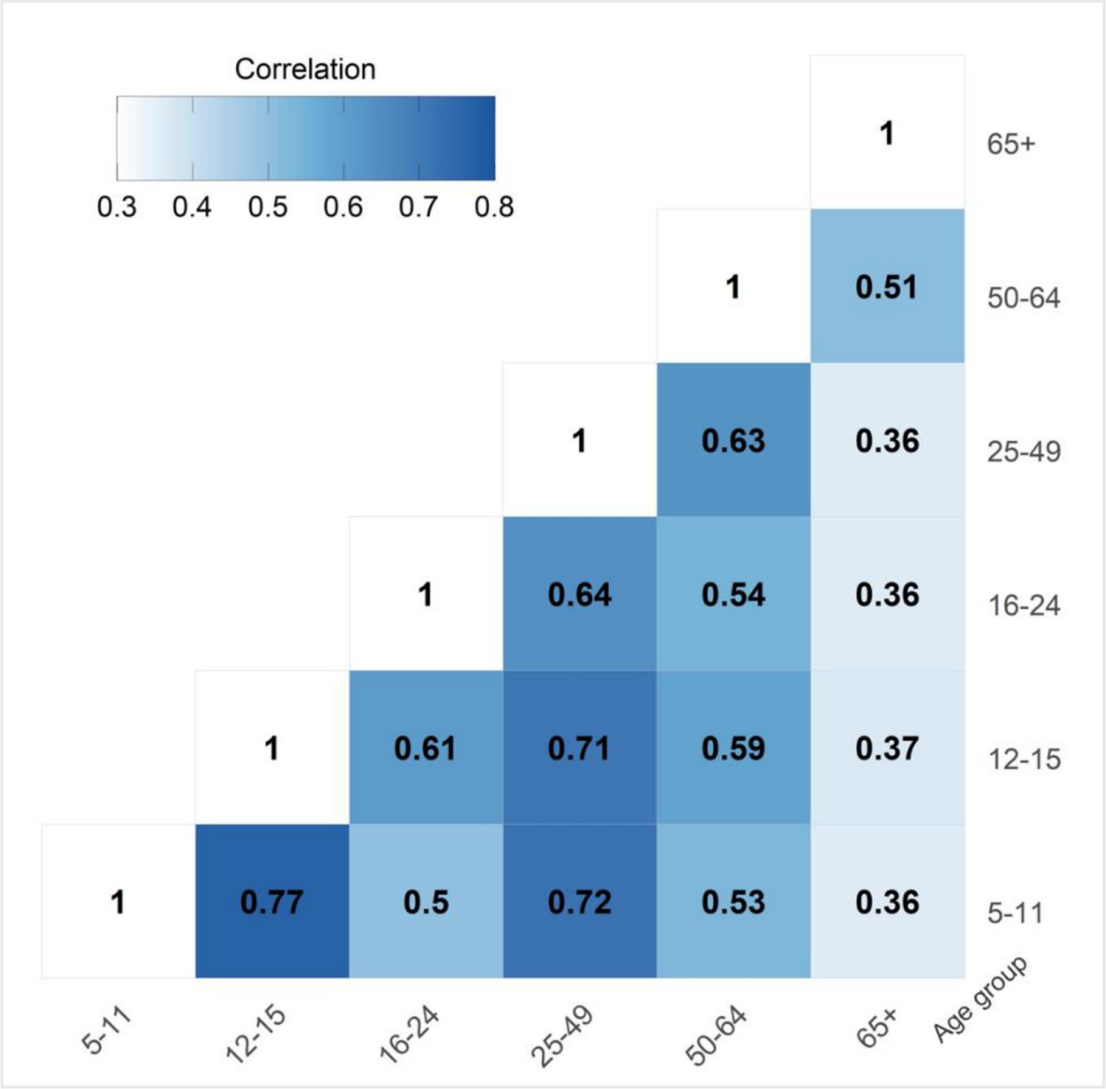
Correlation between age-specific geographic patterns of COVID-19 vaccine completion by Zip code.

Zip code-level vaccine completion percentages for all age groups were positively associated among each other (*p* < 0.001); correlation coefficients ranged from 0.36 to 0.77 (Fig 3). The strongest correlation was between the age 5–11 and 12–15 groups (0.77), followed by the age 5–11 and 25–49 groups (0.72), and age 12–15 and 25–49 groups (0.71). The weakest correlations were with the age 65+ group. Overall, correlations were strongest between adjacent age groups.

### 3.3 Sociodemographic characteristics and vaccination completion

Median household income was positively associated (p < 0.05) with COVID-19 vaccine completion percentage in all unadjusted models (Table 2). IRR rurality score was negatively associated with completion in all unadjusted models. Percent Black population had a significant negative association with completion in the age 5–11 model. For control variables, percent female population was positively associated with higher vaccine completion in all models, while percent healthcare worker population was positively associated with completion in the age 65+ model.

**Table 2:** Regression coefficients of Zip code-level COVID-19 vaccine completion in unadjusted and adjusted spatial lag autoregressive models.

	Age Group (years)						
	Overall	5–11	12–15	16–24	25–49	50–64	65+
**Unadjusted**							
**% Black population**	-0.043	-0.067 *	0.017	-0.051	0.019	0.004	-0.023
**% Female population**	0.199 ***	0.063 *	0.083 **	0.146 ***	0.171 ***	0.194 ***	0.097 **
**Median Household Income**	0.265 ***	0.248 ***	0.213 ***	0.328 ***	0.200 ***	0.230 ***	0.386 ***
**% Healthcare Worker population**	-0.043	0.010	0.005	-0.029	-0.023	-0.005	0.118 ***
**Index of Relative Rurality**	-0.082 *	-0.100 ***	-0.141 ***	-0.102 **	-0.145 ***	-0.184 ***	-0.204 ***
**Adjusted**							
**Spatial lag (ρ)**	0.419 ***	0.540 ***	0.485 ***	0.392 ***	0.452 ***	0.251 ***	NA
**% Black population**	0.044	0.003	0.087 **	0.055	0.081 *	0.069 *	0.086 *
**% Female population**	0.222 ***	0.074 **	0.084 **	0.166 ***	0.176 ***	0.195 ***	0.095 **
**Median Household Income**	0.322 ***	0.255 ***	0.234 ***	0.379 ***	0.228 ***	0.241 ***	0.393 **
**% Healthcare Worker population**	-0.083 **	-0.024	-0.047	-0.071 *	-0.083 **	-0.077 *	0.053
**Index of Relative Rurality**	0.048	-0.0004	-0.054	0.040	-0.062	-0.093 *	-0.027
**Nagelkerke R-squared**	0.312	0.366	0.311	0.322	0.316	0.202	0.167
**AIC**	1877.9	1815.7	1878.4	1866.9	1873.2	1990.2	1964.1

* p < .05

** p ≤ .01

*** p ≤ .001

*Note*. For the unadjusted 65+ median household income model, the spatial lag model was not appropriate, thus the OLS regression results are reported for this cell. Additionally, for the adjusted 65+ model, OLS regression results were also reported in the table.

In the adjusted models, median household income had the strongest association (positive) with vaccine completion percentage (Table 2). Percent Black population was positively associated with completion in four out of seven adjusted models, while rurality was only significant (negative association) in the age 50–64 model. Control variable percent female population remained positively associated with higher vaccine completion in all adjusted models, and percent healthcare worker population was negatively associated with completion in four out of seven models. The spatial lag parameter, rho (ρ), was positive and statistically significant in all adjusted spatial autoregressive models. The age 5–11 model had the best model fit (Nagelkerke R^2^ = 0.37 and AIC = 1815.7), while the age 50–64 and 65+ models had the lowest (50–64: Nagelkerke R^2^ = 0.20 and AIC = 1990.2; 65+: Nagelkerke R^2^ = 0.17 and AIC = 1964.1).

In the sensitivity analysis using spatial error regression, the spatial error parameter, lambda (λ), was significant in each model (S3 Appendix). The regression coefficients and p-values were similar to the spatial lag models. No statistically significant coefficients changed direction. Five coefficients were statistically significant (p < 0.05) in the spatial error models but not in the spatial lag models, including one for percent Black population, two for percent healthcare worker population, and two for IRR. The sensitivity analysis produced findings similar to the main analysis.

## 4. Discussion

Geographic patterns of completion percent were generally most similar when comparing proximate age groups, especially in the younger groups. Clustering of completion percent was generally stronger in the younger age groups but present for all. Despite some differences among the age-specific patterns, vaccine completion was consistently higher in urban areas than in rural Zip codes. The most concerning result was the overlapping low completion clusters in rural Zip codes, which may continue to be at risk for future outbreaks.

At both an area- and individual-level, income has a positive association with COVID-19 vaccination uptake [19–21]. We found that median household income has a strong, positive association with vaccine completion across all age groups (in both unadjusted and adjusted models) at the Zip code-level. Higher income is often an indicator of better health care access, higher education, and more reliable transportation.

In adjusted models, percent Black population was positively associated with vaccine completion in some age groups. This is notable considering that in individual-level studies, people of color have been found to have lower vaccine uptake due to initial roll-out disadvantages, less frequent interaction with healthcare professionals, historical and ongoing medical mistreatment, cost-related concerns, and poorly executed promotional efforts [15]. Prior to COVID-19, North Carolina worked to decrease racial and ethnic disparities and promote vaccination equity. Furthermore, during the pandemic, the CDC ranked the state in the top 10 for equitable vaccine coverage [45]. Specifically, reports note that the state’s equity strategies promoted vaccination among Black and Hispanic communities [46]. Although North Carolina has made steps towards decreasing racial and ethnic disparities and promoting vaccine equity, this is still an ongoing effort.

Individual-level studies have found lower COVID-19 vaccination uptake in rural areas [47]. In our analysis, for all age groups, geographic clusters of high vaccine completion were in urban areas. In the unadjusted models, rurality was negatively associated with vaccine completion percent; however, in the adjusted models, rurality was only associated in the age 50–64 model. This is likely due to stronger associations with other factors (such as median household income) that are somewhat collinear with rurality. Factors not considered, such as differences in vaccine accessibility and availability between urban and rural areas may help to explain these differences as well. North Carolina did make attempts to remove transportation barriers to vaccine access through allocating $2.5 million to local transit authorities to offset transportation costs for people to travel to and from vaccine appointments [48].

We included control variables capturing Zip code-level percent female population and percent of population who are healthcare workers. In the adjusted analyses, percent female was significantly positively associated in all models and percent healthcare workers was negatively associated in four out of seven models, suggesting their inclusion was important to estimating the associations with the variables of interest. It was initially hypothesized that percent healthcare worker population would have a positive association with vaccine completion, however the results do not support this hypothesis. Many other researchers have conducted studies on the willingness of healthcare workers to receive the vaccine [49, 50], opening an avenue for future research on this relationship.

Older adults have higher risk of poor outcomes or death from COVID-19 [51], a main reason that they gained early access to the vaccines [11]. As such, they may have been more motivated to get vaccinated [52, 53]. Older North Carolinians had much higher vaccine completion percentages. Early vaccine rollouts included mass vaccination clinics, pop-up clinics, and increased funding, making the vaccine more accessible to groups who were eligible earlier. Some long-term care facilities mandated or strongly recommended residents be vaccinated [54]. High levels of completion in this group may also be due to successful public health messaging and an effective vaccine rollout. Likely, it was a combination of these factors that contributed to substantial differences in both the magnitude and geographic patterns of vaccine completion for the older population in North Carolina.

COVID-19 vaccine uptake for children is strongly associated with their parents’ intentions to get vaccinated themselves [55]. At the area-level, we observed strong, positive correlation between completion percentages in the age 5–11 and 25–49 groups, and in the age 12–15 and 25–49 groups. These findings may be due to the parent/child relationship of Zip code residents in these age groups, suggesting that one approach to increase completion in the younger population is by promoting completion in the parent population. Regardless, vaccination for children may continue to be low considering these groups did not receive the benefits of early vaccination distribution (e.g. mass vaccination clinics).

Although this study focuses on completion of the primary vaccine series, booster shots were made available in September 2021 for adults at least six months out from having completed the primary series [56]. Due to waning immunity of the primary series [57] and the emergence of new variants, booster shots have been strongly recommended. Those who have researched willingness to accept a booster shot have found a strong association with acceptance of the primary vaccination series [58]. Thus, this analysis on primary vaccine completion can also provide meaningful insights for booster vaccine uptake.

### 4.1 Limitations

When using aggregated data at any geographic scale, we should be cautious about assuming the findings are valid at an individual-level (the ecological fallacy [59]). Given that individual-level data were unavailable for all sociodemographic characteristics we were interested in, our study used an ecological design. Although inferences about individual’s vaccination decisions cannot be made from our study, we believe it to be an important contribution to COVID-19 vaccine uptake research.

Zip code boundaries are not congruent with U.S. Census enumeration unit boundaries, requiring areal interpolation to estimate their demographic and socioeconomic attributes and introducing the potential for error due to this process. Other comparable approaches to estimate Zip code-level data using Census data are available [60]. Zip code boundaries also change quite frequently; given the relatively short time period of this study, this likely did not affect our findings. Further, we were unable to conduct our analysis at additional spatial scales because the base unit was a Zip code, thus our findings may be limited to this specific geographic resolution of the data. However, the CVMS data were reported by Zip code, which did not require geocoding and provided a relatively high spatial resolution.

The data available at the time of analysis only had vaccination information through mid-2022. Because people became eligible for the COVID-19 vaccine at varying time points based on age, one concern was that the data for the younger age groups was not reflective of eventual levels of completion. We compared overall statewide levels to data available by NCDHHS during April 2023 and found that the younger age groups had little increase in uptake during this period [61]. This likely did not affect our results but is worth exploring further in future research. Furthermore, our results are only internally valid and not generalizable to other timeframes, states, countries, or vaccines.

Finally, we recognize that rurality does not have a single definition. Our analysis used the IRR, but other documented methods of measuring rurality are worth exploring further.

## 5. Conclusion

Limited research has simultaneously examined COVID-19 vaccine uptake at fine levels of geographic resolution and across age groups. Understanding where and why both high and low vaccination uptake is clustered is necessary information for controlling disease spread and implementing interventions. This work can inform both geographic-targeting and age-specific targeting of vaccine initiatives.

## Supporting information

S1 Appendix Processing vaccination data from NCDHHS. (DOCX)

S2 Appendix Education, political affiliation, and ethnicity variables. (DOCX)

S3 Appendix Sensitivity analysis. (DOCX)
