# Supplementary material for: The geography of COVID-19 vaccine completion by age in North Carolina, U.S
Source: PLoS One. 2024 Aug 9;19(8):e0304812. doi: 10.1371/journal.pone.0304812 (PMC11315330; doi:10.1371/journal.pone.0304812)
Supplement: S1 Appendix — (DOCX) [file pone.0304812.s001.docx]

**S1 Appendix: Processing Vaccination Data from NCDHHS**

The vaccination data were extracted from records in COVID-19 Vaccine Management System (CVMS) supplied by the North Carolina Department of Health and Human Services, which included all COVID-19 vaccines administered in North Carolina. The CVMS is dose-level database that include a unique identifier for each person, as well as each individual’s age, race, gender, and zip code of residence at time of vaccination. The CVMS data used for analysis include all vaccination doses from the beginning of rollout (late 2020) to May 31, 2022 (the most recent available data at the time of analysis).

The raw CVMS data included 18,335,539 records. We removed records using a stepwise process in the following order, 1) did not have a unique patient ID (n = 164), 2) were missing Zip Code information (n = 21,322), 3) had a non-NC or unidentifiable Zip Code (n = 645,122), and/or 4) were flagged as a duplicate record (had the same patient ID, Date, Zip Code, and vaccine manufacturer, n = 2,020,858); the large number of duplicated records were due to the raw data delivery method (an initial large set of records and subsequent updates which included duplicate records from prior update files). After removals, 15,669,412 records remained.

The CVMS included a “dose number” variable that appeared to be unreliable for determining a person’s vaccination status for two reasons, 1) the number of doses to complete vaccination for the mRNA vaccines (2 doses) was different for the Janssen vaccine (1 dose) and 2) many people received doses from different manufacturers. Therefore, we developed an algorithm to leverage patient ID, dose number, date, and vaccine type to determine what type of dose was received, which included initial, complete, boosted, boosted plus (received more than one booster dose). The algorithm is provided below based on the date and manufacturer:

- Initial Dose
  - First dose is an mRNA vaccine
- Completion Dose
  - Second dose is an mRNA vaccine for people who had received an mRNA vaccine for their first dose
  - First dose is Janssen
  - Second dose is Janssen for people who had received an mRNA vaccine for their first dose
- Booster Dose
  - Third dose is an mRNA vaccine for people who had received mRNA vaccines for their first two doses
  - Second dose is an mRNA vaccine for people who had received Janssen for their first dose
  - Third dose is an mRNA vaccine for people who had received an mRNA vaccine for their first dose and Janssen for their second dose
  - Second dose is Janssen for people who had received Janssen for their first dose
  - Third dose is Janssen for people who had received mRNA vaccines for their first two doses
  - Third dose is Janssen for people who had received an mRNA vaccine for their first dose and Janssen for their second dose
- Booster Plus Dose
  - Fourth dose is an mRNA vaccine for people who had received mRNA vaccines for their first three doses
  - Third dose is an mRNA vaccine for people who had received Janssen for their first dose and an mRNA vaccine for their second dose
  - Fourth dose is an mRNA vaccine for people who had received an mRNA vaccine for their first dose, Janssen for their second dose, and an mRNA vaccine for their third dose
  - Third dose is Janssen for people who had received Janssen for their first two doses
  - Fourth dose is Janssen for people who had received mRNA vaccines for their first two doses and Janssen for their third dose
  - Fourth dose is Janssen for people who had received an mRNA vaccine for their first dose and Janssen for their second and third doses

Using the above algorithm, 5,923,984 doses were identified as initial, 6,038,423 were identified as completion, 3,461,288 were identified as booster, and 245,717 were identified as booster plus.

Based on the age of the vaccine recipient, we assigned each record an age category (0-4, 5-11, 12-15, 16-24, 25-49, 50-64, and 65+ years). We then subset the records to only include individuals who had received completion doses and aggregated count values by Zip Code and age category.
