# Supplementary material for: The geography of COVID-19 vaccine completion by age in North Carolina, U.S
Source: PLoS One. 2024 Aug 9;19(8):e0304812. doi: 10.1371/journal.pone.0304812 (PMC11315330; doi:10.1371/journal.pone.0304812)
Supplement: S2 Appendix — (DOCX) [file pone.0304812.s002.docx]

**S2 Appendix: Education, political affiliation, and ethnicity variables**

Like the other sociodemographic variables, education and ethnicity data came from the U.S. Census Bureau’s American Community Survey (ACS) 5-year estimate for 2016-2020 [23]. Both the education (% population with any higher education) and ethnicity (% Hispanic population) underwent the same cleaning processes as the other variables collected from the ACS. However, education proportions were based on the population aged 25+.

Voter registration data for North Carolina came from the NC State Board of Elections, which included the registered party and Zip code of currently registered voters in the state as of November 5, 2022 [60]. For political affiliation (% registered Democrat population), we used a table join to join the voter registration data to the Zip code crosswalk table and then aggregated the count of people registered for each political party and the total count of registered voters by each Zip code. This count data was used to calculate the proportion of people in each political party for each Zip code.

Although education is an interesting and important variable associated with COVID-19 vaccine uptake, we found it to be highly correlated with income *and* rurality (S2 Table). In order to keep education in the model, we would have had to forfeit examining income and rurality. Additionally, political affiliation was highly correlated with race, and ethnicity was highly correlated with rurality, however we assumed race and rurality to be more important variables in investigating our research goals. Given this information, we chose to exclude education, political affiliation, and ethnicity from the final regression analyses.

***S2 Table.* Pearson’s correlation coefficients between all potential predictor variables**

| Variable | Median Household Income | % Education > high school | % Black population | % Female population | % Health Care Worker population | % Registered Democrat population | Index of Relative Rurality |
| --- | --- | --- | --- | --- | --- | --- | --- |
| Median Household Income |  |  |  |  |  |  |  |
| % Education > high school | 0.757 |  | | | | | |
| % Black population | -0.304 | -0.266 |  | | | | |
| % Female  population | -0.005 | 0.092 | 0.060 |  | | | |
| % Healthcare Worker population | 0.146 | 0.243 | 0.077 | 0.074 |  | | |
| % Registered Democrat population | -0.235 | -0.120 | 0.867 | 0.104 | 0.134 |  | |
| Index of Relative Rurality | -0.462 | -0.592 | -0.043 | -0.127 | -0.335 | -0.084 |  |
| % Hispanic population | 0.183 | 0.203 | 0.197 | 0.070 | 0.255 | 0.203 | -0.586 |
