# Supplementary material for: The geography of COVID-19 vaccine completion by age in North Carolina, U.S
Source: PLoS One. 2024 Aug 9;19(8):e0304812. doi: 10.1371/journal.pone.0304812 (PMC11315330; doi:10.1371/journal.pone.0304812)
Supplement: S3 Appendix — (DOCX) [file pone.0304812.s003.docx]

# S3 Appendix: Sensitivity analysis

***S3 Table.* Regression coefficients of Zip code-level COVID-19 vaccine completion in adjusted spatial error autoregressive models**

|  | Age Group (years) | | | | | | |
| --- | --- | --- | --- | --- | --- | --- | --- |
|  | **Overall** | **5-11** | **12-15** | **16-24** | **25-49** | **50-64** | **65+** |
| Spatial error (λ) | 0.429 *** | 0.559 *** | 0.492 *** | 0.394 *** | 0.461 *** | 0.236 *** | NA |
| % Black population | 0.074 | -0.0005 | 0.130 ** | 0.089 * | 0.127 ** | 0.084 * | 0.086 * |
| % Female  population | 0.215 *** | 0.070 * | 0.082 ** | 0.166 *** | 0.168 *** | 0.197 *** | 0.095 ** |
| Median Household Income | 0.379 *** | 0.311 *** | 0.291 *** | 0.441 *** | 0.283 *** | 0.261 *** | 0.393 ** |
| % Healthcare Worker  population | -0.108 ** | -0.062 * | -0.076 * | -0.097 ** | -0.109 *** | -0.093 ** | 0.053 |
| Index of Relative Rurality | 0.005 | -0.073 | -0.127 * | 0.002 | -0.119 * | -0.142 ** | -0.027 |
| Nagelkerke R-squared | 0.302 | 0.360 | 0.304 | 0.310 | 0.306 | 0.195 | 0.167 |
| AIC | 1888.6 | 1822.6 | 1886.5 | 1880.4 | 1884.7 | 1996.2 | 1964.1 |

* p < .05, ** p ≤ .01, *** p ≤ .001

*Note.* The table shows results from the spatial error models with the exception of the 65+ age group. OLS regression was used for the 65+ age group as the spatial error model was not statistically significant for this group.
